# Outbreak Investigation of *Pseudomonas aeruginosa* Endophthalmitis Following Cataract Surgery in a Private Ophthalmology Clinic in Korea

**DOI:** 10.3390/pathogens15050480

**Published:** 2026-04-29

**Authors:** Min A Lim, Na Young Hong, Sook Hee Park, Myung Hee Kim, Youkyoung Kim, Ji Hong Park, Dong Gyu Park, Hee Young Hwang, Seok Ju Yoo, Ji Hyuk Park, Kwan Lee

**Affiliations:** 1 Department of Preventive Medicine, College of Medicine, Dongguk University, Gyeongju 38066, Republic of Korea; minadid@korea.kr (M.A.L.); gbcidc5@gbcidc.or.kr (N.Y.H.); medhippo@dongguk.ac.kr (S.J.Y.); skeyd@dongguk.ac.kr (J.H.P.); 2 Division of Public Medicine, Gyeongsangbuk-do Provincial Government, Andong 36759, Republic of Korea; bergamot77@korea.kr (M.H.K.); dbruddl9975@korea.kr (Y.K.); jihongp@korea.kr (J.H.P.); 3 Gyeongbuk Center for Infectious Diseases Control and Prevention, Andong 36759, Republic of Korea; gbcidc4@gbcidc.or.kr; 4 Division of Infectious Disease Research, Gyeongsangbuk-do Health & Environment Research Center, Yeongcheon 38874, Republic of Korea; water77@korea.kr (D.G.P.); hhy0619@korea.kr (H.Y.H.)

**Keywords:** cataract surgery, endophthalmitis, *Pseudomonas aeruginosa*, environmental sampling, infection control

## Abstract

Postoperative endophthalmitis is a rare but severe complication of cataract surgery that may lead to significant visual impairment and can occasionally present as clusters or outbreaks linked to lapses in infection control. On 29 October 2025, three cases of postoperative endophthalmitis following cataract surgery at an ophthalmology clinic (Clinic A) in Yeongju, Republic of Korea, were reported to public health authorities. All cases were confirmed as *Pseudomonas aeruginosa* (*P. aeruginosa*). An epidemiological investigation was conducted to identify the source and transmission route; all 54 patients who underwent cataract surgery at Clinic A in October 2025 (62 procedures, including eight bilateral cases) were included. Infection control practices were assessed through on-site inspection, staff interviews, medical record review, and telephone surveys. Environmental samples were collected and cultured selectively for *P. aeruginosa*. Isolates were analyzed using pulsed-field gel electrophoresis (PFGE) to assess genetic relatedness. Among 54 surgical patients, three developed endophthalmitis (attack rate: 5.6%). All cases occurred in patients operated on 23 October by the same surgeon; no additional cases were identified. *P. aeruginosa* was isolated from all three patients. Of 45 environmental samples, three were positive: the laundry room drain, the sink drain in the local anesthesia room, and the interior of cassette No. 2 and instruments within the operating room steam sterilizer. PFGE showed 95.7% band pattern similarity between patient isolates and those from the sterilizer. This outbreak was attributed to *P. aeruginosa*, with the steam sterilizer identified as the most probable source. Inadequate infection control and sterilization practices likely contributed. These findings highlight the critical importance of strict sterilization monitoring and adherence to infection prevention and control practices to prevent recurrence.

## 1. Introduction

Cataract surgery is one of the most commonly performed surgical procedures worldwide. Although the incidence of postoperative endophthalmitis is low and has drastically reduced during the most recent decades [1], it remains a serious complication that may result in profound visual impairment or permanent vision loss. The reported incidence of acute postoperative endophthalmitis ranges from 0.03% to 0.2% [2,3,4]. Most cases are bacterial in origin [5]. In a Korean study analyzing 113 patients with endophthalmitis, *Staphylococcus epidermidis* was the most frequently identified pathogen, followed by *Pseudomonas aeruginosa* (*P. aeruginosa*) [6].

*P. aeruginosa* is traditionally classified as an opportunistic pathogen [7]; however, in certain clinical contexts such as postoperative endophthalmitis, it can act as a highly virulent organism capable of causing severe and rapidly progressive infection even in otherwise healthy individuals [8]. It can be transmitted through contaminated medical devices, irrigation fluids, disinfectants, and other components of the healthcare environment. Consequently, the level of infection prevention and control within medical facilities plays a critical role in preventing outbreaks [9,10].

Post-cataract endophthalmitis caused by *P. aeruginosa* is known to present a more rapid and aggressive clinical course compared with other bacterial pathogens [11,12]. Due to its potent virulence factors and tissue invasiveness, inflammation can progress within hours to days after infection onset. Severe clinical manifestations—including intense ocular pain, hypopyon, corneal opacity, and hypotony—are frequently observed. Moreover, the organism often exhibits multidrug resistance and the capacity to form biofilms, contributing to treatment failure. Delayed or inadequate management may lead to irreversible visual loss or even enucleation [13,14,15]. In addition to postoperative infections, *P. aeruginosa* is also a recognized cause of endogenous endophthalmitis, in which hematogenous spread can result in severe intraocular infection, particularly in patients with systemic risk factors [16].

Outbreaks of postoperative endophthalmitis have also been reported in Korea. In September 2020, an investigation involving 156 cataract surgery patients at a single ophthalmology clinic identified genetically identical *Fusarium oxysporum* isolates in both patients and a surgical material (viscoelastic substance) [17,18]. The mean incubation period was approximately 24 days, consistent with the clinical course of fungal endophthalmitis. This event underscored the potential for contaminated surgical materials to cause healthcare-associated outbreaks and highlighted the importance of stringent infection control and quality assurance measures.

The present outbreak at Clinic A was first recognized on 29 October 2025, following a complaint reported to the Yeongju Public Health Center. On 30 October, an initial investigation confirmed that three of four patients who had undergone cataract surgery on October 23 by the same surgeon developed postoperative endophthalmitis. This study aimed to investigate an outbreak of postoperative endophthalmitis at a private ophthalmology clinic, identify the source and transmission route, and propose appropriate infection control measures.

## 2. Materials and Methods

### 2.1. Composition and Roles of the Epidemiological Investigation Team

The outbreak investigation and response were conducted collaboratively by the Gyeongsangbuk-do Provincial Government, the Yeongju Public Health Center, the Gyeongsangbuk-do Infectious Disease Control and Prevention Support Group, the Korea Disease Control and Prevention Agency (KDCA), and the Gyeongsangbuk-do Medical Association. The Provincial Government and the Infectious Disease Control and Prevention Support Group coordinated the overall case investigation, reviewed medical records, and supported the field epidemiological assessment. The Yeongju Public Health Center conducted telephone surveys of surgical patients and managed administrative procedures. The KDCA provided consultation on the design of the epidemiological investigation and performed molecular analyses of the identified pathogens. The Gyeongsangbuk-do Medical Association provided expert consultation on environmental remediation and strengthening infection prevention and control measures, based on current evidence in cataract surgery and the findings of the investigation.

### 2.2. Methods

#### 2.2.1. Case Definition and Case-Series Investigation

This study was a retrospective cohort-based outbreak investigation with a descriptive case-series component. A case was defined as any patient who developed postoperative endophthalmitis (including ocular pain, decreased vision, hypopyon, or other signs of intraocular inflammation) after cataract surgery performed at Clinic A (Yeongju-si, Republic of Korea) between 1 October and 31 October 2025, with microbiological confirmation, regardless of whether the diagnosis was made at Clinic A or at another healthcare facility after referral. No probable cases were included in the final analysis. A total of 62 cataract procedures were performed during the study period, including eight bilateral surgeries, resulting in 54 unique patients. Attack rates were calculated using the number of patients as the denominator.

A field epidemiologic outbreak investigation with a retrospective cohort component design was applied to identify potential causes and transmission pathways. On 30 October 2025, the Yeongju Public Health Center conducted the initial investigation and secured the list of surgical patients and corresponding medical records from 1 October onward. Demographic characteristics, dates of clinic visits, surgical dates, and the occurrence of postoperative endophthalmitis were reviewed. Among the 54 surgical patients, 45 were successfully contacted, excluding nine who were unreachable. Telephone interviews were conducted using a standardized questionnaire that included key symptoms of endophthalmitis (e.g., ocular pain, conjunctival injection, decreased visual acuity, photophobia, periorbital swelling, and ocular discharge) and information regarding visits to other medical institutions. Thus, the study population included all patients who underwent cataract surgery at Clinic A during October 2025. The primary outcome was postoperative endophthalmitis. The primary exposure of interest was the date of surgery and potential exposure to contaminated surgical instruments processed in the steam sterilizer.

#### 2.2.2. On-Site Inspection and Procedural Reenactment

During the second investigation on 31 October 2025, cataract surgeries at the facility were suspended following recommendations from the KDCA. On 3 November, a third on-site inspection was conducted with participation from the Provincial Government, the Yeongju Public Health Center, and the Infectious Disease Control and Prevention Support Group. Staff interviews and a reenactment of the surgical process were performed to identify potential risk factors. Individual interviews were conducted with staff members in a nurse changing room located in front of the second-floor operating room. Each staff member described in detail their responsibilities before, during, and after surgery, including medication management, infection control practices, and environmental cleaning procedures. The same processes were subsequently reenacted at their actual workstations, and the procedures were documented via video recording.

#### 2.2.3. Laboratory Investigation

After the onset of endophthalmitis, the three confirmed cases were transferred from Clinic A to a university hospital in a nearby metropolitan city. Ocular specimens collected at the referral hospital yielded *P. aeruginosa*. An assessment of infection control practices was conducted across outpatient clinics, operating rooms, sterilization areas, and laundry facilities. A total of 45 environmental samples were collected from potentially contaminated instruments and environmental surfaces using One Touch Media transport swabs (Table 1). In addition, specimens were obtained from the fingertips of both hands of four healthcare personnel directly involved in cataract surgeries (out of nine total staff members), yielding four human specimens.

A total of 49 specimens (45 environmental and 4 human) were cultured at the Gyeongsangbuk-do Institute of Health and Environment. Selective culturing was performed to identify isolates consistent with *P. aeruginosa* detected in the three case patients. Samples were streaked onto Tryptic Soy Agar (TSA) plates for microbial identification. Sampling sites were selected based on potential contamination points identified during the initial field inspection. Among the environmental specimens, three samples—from the laundry room drain, the sink drain in the local anesthesia room, and the operating room steam sterilizer STATIM 2000 G4^®^ (SciCan Ltd., Toronto, ON, Canada)—were included. To determine genetic relatedness between isolates from patients and environmental samples, pulsed-field gel electrophoresis (PFGE) was performed by the Bacterial Analysis Division of the KDCA. According to established PFGE interpretation criteria [19], isolates with ≥95% similarity are considered closely related.

#### 2.2.4. Data Analysis

This study was conducted as a field epidemiologic investigation of an outbreak within a defined surgical cohort. Descriptive analyses were performed to characterize cases, calculate attack rates, and assess temporal clustering. Owing to the small sample size and absence of a comparison group, inferential statistical testing was not considered appropriate.

#### 2.2.5. Ethics

This study was conducted as part of a legally mandated public health investigation under the Infectious Disease Control and Prevention Act of the Republic of Korea. Therefore, separate review and approval by an Institutional Review Board (IRB) or Ethics Committee were not required. All personal identifiers were removed prior to data analysis.

Prior to participation, individuals were informed of the purpose and procedures of the epidemiological investigation. Written informed consent was not required for this type of legally mandated public health investigation; however, verbal informed consent was obtained in accordance with applicable regulations and standard public health practice.

## 3. Results

### 3.1. Characteristics of Patients

During the one-month investigation period, a total of 62 cataract surgeries (54 individuals) were performed at Clinic A. Postoperative endophthalmitis occurred in 3 patients, corresponding to an attack rate of 5.6% based on a patient-level denominator. Among the four patients who underwent surgery on 23 October, three developed endophthalmitis (attack rate: 75%). All three patients were male: two were in their 70s and one was in his 50s. All underwent left-eye cataract surgery on 23 October 2025, performed by the same surgeon. Each patient returned to the clinic the following day, 24 October, and was diagnosed with postoperative endophthalmitis. No additional cases were identified thereafter. All affected patients were transferred to a university hospital in a nearby metropolitan city for further management.

All patients received postoperative prophylactic antibiotics, including topical levofloxacin (Lextacin^®^ ophthalmic solution) administered immediately after surgery and oral cefaclor (250 mg, three times daily).

### 3.2. Healthcare Personnel and Facility Characteristics

**Figure 1:**
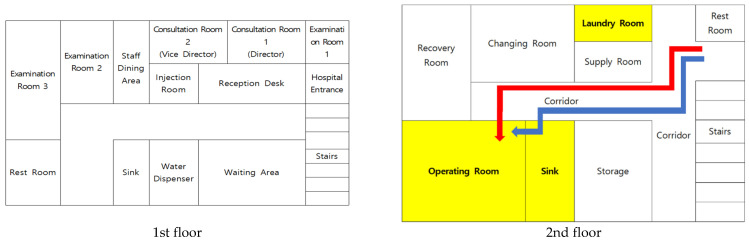
Layout of Clinic A (**■** healthcare personnel pathway; **■** patient pathway; **■** locations where *P. aeruginosa* was detected).

Clinic A employed two board-certified ophthalmologists, one registered nurse, and six nurse assistants. Routine cleaning tasks were performed by a part-time worker. The outpatient clinic and diagnostic areas were located on the first floor, while the operating room was situated on the second floor, providing spatial separation between clinical and surgical areas (Figure 1). A total of four personnel participated in cataract surgeries, including two ophthalmologists, one registered nurse, and one nurse assistant. Human specimens for microbiological testing were collected from all four surgical staff members. The clinic managed approximately 100 outpatient visits per day and performed an average of three to four surgeries daily. Surgical schedules were not fixed; when same-day surgery was deemed necessary, procedures could be performed immediately if the medical staff’s schedule permitted.

### 3.3. Site Inspection and Procedural Reenactment

#### 3.3.1. Assessment of Infection Prevention and Control Practices

**Figure 2:**
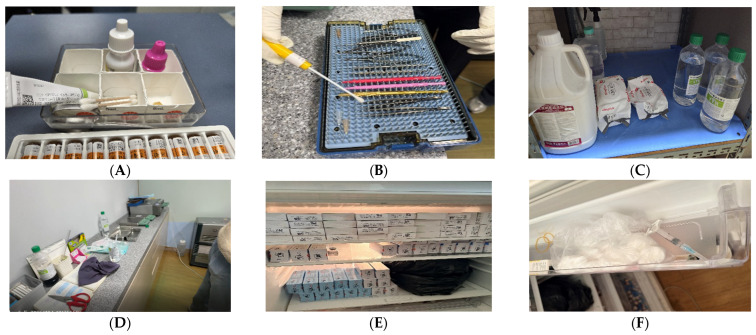
Representative observations related to infection prevention and control practices at Clinic A. (**A**) Use and handling of ophthalmic medications, (**B**) processing and reuse of surgical materials, (**C**) management of water used in sterilization processes, (**D**) spatial organization of clean and contaminated areas, (**E**) storage conditions of medical supplies, and (**F**) handling of used medical devices.

During the on-site inspection, multiple deficiencies in infection prevention and control were identified (Figure 2). These included deviations from recommended practices in the handling and use of ophthalmic medications and surgical materials, as well as inconsistencies in the management of supplies intended for single use.

In addition, practices related to water use and instrument processing showed variability, including the handling of fluids associated with cleaning and sterilization processes. Within the operating room, spatial separation between patient care areas and instrument processing areas was not clearly delineated, resulting in overlap between clean and potentially contaminated zones.

No formal written guidelines or standardized protocols were available for the disinfection and management of ophthalmic instruments and diagnostic equipment. Environmental cleaning and disinfection practices across outpatient, operating, and preparation areas were not consistently implemented. Storage and handling practices for medical supplies and related materials also demonstrated inconsistencies, suggesting gaps in adherence to standard infection prevention and control procedures. Overall, these findings indicate systemic weaknesses in infection control practices rather than isolated issues.

#### 3.3.2. Environmental Management

**Figure 3:**
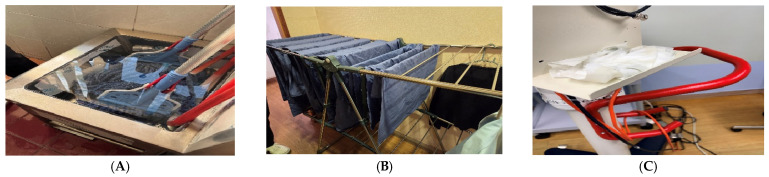
Representative observations related to environmental management and cleaning practices at Clinic A. (**A**) Handling and storage of cleaning equipment, (**B**) laundering and drying of surgical textiles, and (**C**) management of used materials following procedures.

Environmental cleaning activities were conducted by a part-time worker and were primarily focused on general areas such as floors and restrooms (Figure 3). The use of detergents and disinfectants was not consistently standardized, and variations in cleaning practices across different areas were observed. Disinfection of medical equipment and environmental surfaces was not routinely or consistently implemented. Medical textiles, including surgical gowns and drapes, were laundered on-site and dried in areas adjacent to the operating room. Variability in drying and subsequent handling processes was noted, which may have implications for effective decontamination and microbial control.

Overall, these observations suggest inconsistencies in environmental cleaning and textile management practices, potentially contributing to increased risk of environmental contamination.

#### 3.3.3. Interviews with Healthcare Personnel and Procedural Reenactment

During October 2025, a total of 62 cataract surgeries were performed by two ophthalmologists. Separate instrument sets were used, and due to the short intervals between procedures, instruments were processed using a short-cycle steam sterilizer. Interviews and procedural reenactment identified several inconsistencies in perioperative practices. Surgical staff were observed to enter the operating room without consistently changing into dedicated surgical attire. Although preoperative hand hygiene was reportedly performed, variations in hand preparation practices and adherence to recommended protocols were noted. During the operative process itself, aseptic techniques were generally maintained. The operating room environment was managed by designated staff; however, no formal written protocols for cleaning and disinfection were in place. Environmental cleaning practices were performed intermittently, primarily when visible contamination was present. Instrument reprocessing practices showed variability, including limited use of recommended detergents and mechanical cleaning methods prior to sterilization.

**Figure 4:**
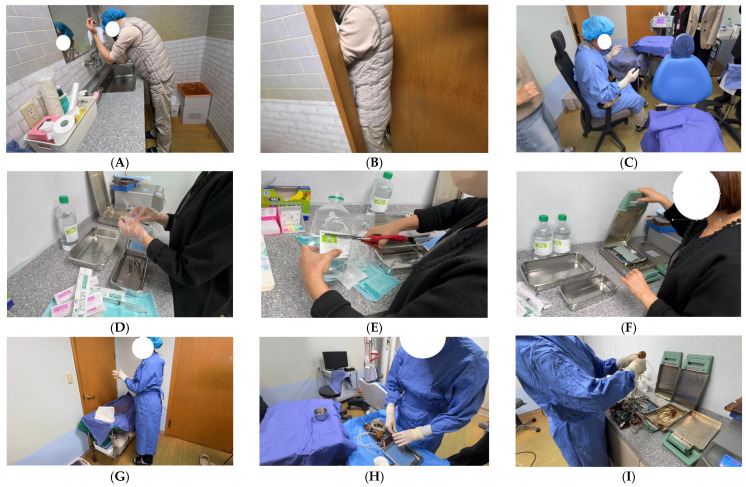
Representative observations from procedural reenactment at Clinic A. (**A**–**I**) Key steps in the surgical workflow, including hand hygiene, preparation practices, instrument handling, sterilization processes, and perioperative procedures.

In addition, sterilization monitoring procedures, including mechanical, chemical, and biological indicators, were not consistently implemented. Delays in instrument reprocessing and variations in handling practices between procedures were also identified, which may have increased the risk of inadequate sterilization. One assistant was involved in operating room support and instrument processing. Formal training in infection prevention and control was limited, and responsibilities related to sterilization processes were performed under supervision. Overall, these findings suggest systemic gaps in infection prevention and control practices rather than isolated deviations (Figure 4).

### 3.4. Laboratory Findings

**Figure 5:**
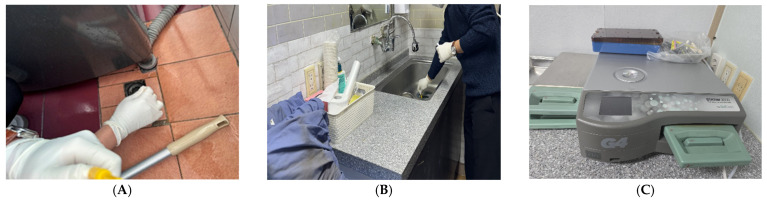
Locations of *P. aeruginosa* Detection. (**A**) Laundry room drain, (**B**) Sink drain in the local anesthesia room, (**C**) Operating room steam sterilizer, cassette No. 2.

*P. aeruginosa* was isolated from all three case patients. However, none of the four healthcare personnel who participated in the surgical procedures tested positive for *P. aeruginosa* in human specimens (Figure 5).

Among the 45 environmental samples collected, three were positive for *P. aeruginosa*. The positive sites included the laundry room drain, the sink drain in the local anesthesia room, and the operating room steam sterilizer, specifically from the interior of cassette No. 2 and associated surgical instruments. All positive environmental samples were obtained from areas located on the second floor.

**Figure 6:**
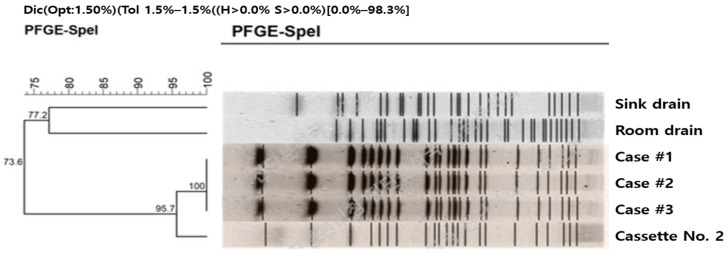
PFGE analysis results of isolates from case patients and environmental samples. A Ko-rean-language watermark indicating the source institution (Department of Laboratory Diagnosis and Analysis, Korea Disease Control and Prevention Agency, KDCA) is present in the original image and has been retained for clarity and attribution.

Genetic relatedness analysis was performed on isolates obtained from the three case patients and the three environmental samples. The isolate from the operating room steam sterilizer (cassette No. 2 and surgical instruments) demonstrated 95.7% band pattern similarity with the patient isolates, indicating a high likelihood of a common source. In contrast, isolates from the sink drain and laundry room drain showed a lower similarity of 73.6% (Figure 6).

## 4. Discussion

This outbreak of postoperative endophthalmitis was temporally and epidemiologically associated with cataract surgeries performed on 23 October 2025, at Clinic A. All three confirmed cases underwent surgery on the same day by the same surgeon, and no additional cases occurred before or after this date. The incubation period of approximately one day observed in all cases is consistent with the well-known rapid clinical progression of *P. aeruginosa* endophthalmitis. Previous studies have reported that acute postoperative endophthalmitis caused by *P. aeruginosa* typically presents within 24–72 h following surgery due to the organism’s high virulence and rapid intraocular proliferation [20,21].

Microbiological findings strongly support a common source of infection. *P. aeruginosa* was isolated from all three patients and from three environmental samples, including the laundry room drain, the sink drain in the local anesthesia room, and the interior of cassette No. 2 and associated instruments in the operating room steam sterilizer. Molecular typing using pulsed-field gel electrophoresis demonstrated a high genetic similarity (95.7%) between isolates from patients and those recovered from the sterilizer. According to established PFGE interpretation criteria, isolates with ≥95% similarity are considered closely related and suggest a common source of contamination. In contrast, isolates from the sink and laundry room drains showed lower similarity (73.6%), indicating that these sites may have served as environmental reservoirs rather than the direct source of infection.

The epidemiological pattern of clustering on a single surgical day, combined with molecular evidence, strongly suggests that contaminated surgical instruments processed in the sterilizer represented the most plausible vehicle of transmission. Although several deficiencies in infection prevention and control were identified throughout the facility, the concentration of cases on 23 October indicates a probable point-source exposure. One possible explanation is that bacterial contamination or biofilm accumulation within the sterilizer system reached a threshold level sufficient to contaminate instruments used during that day’s procedures. Alternatively, instruments that had not been adequately cleaned from previous surgical sessions may have introduced microbial contamination into the sterilization cycle.

These findings are particularly important given the pathogenic characteristics of *P. aeruginosa.* In addition to its virulence, antimicrobial resistance further complicates the management of *P. aeruginosa* infections. Given the well-recognized multidrug resistance potential of *P. aeruginosa*, the effectiveness of standard prophylactic antibiotic regimens may be limited. *P. aeruginosa* has demonstrated resistance to commonly used antimicrobial agents, including certain fluoroquinolones and cephalosporins, which raises concerns regarding the adequacy of routine postoperative prophylaxis in preventing infection [22,23]. This highlights the importance of infection prevention and control measures over reliance on antimicrobial prophylaxis alone, particularly in settings where resistant organisms may be present.

Interviews with healthcare personnel and procedural reenactment revealed substantial deficiencies in instrument reprocessing. Surgical instruments were inadequately cleaned and were briefly rinsed in sterile distilled water or flushed using a syringe without the use of detergents or appropriate mechanical cleaning tools. Such practices deviate from recommended guidelines for ophthalmic instrument reprocessing [24]. Ophthalmic surgical instruments often contain complex microstructures and lumens in which organic debris can accumulate. If these residues are not effectively removed prior to sterilization, they may protect microorganisms from heat penetration and promote microbial survival.

Short-cycle steam sterilizers such as the STATIM 2000 G4^®^ system are commonly used in ophthalmic surgical settings because they allow for rapid instrument turnover between procedures. However, proper operation of these devices requires strict adherence to sterilization monitoring protocols, including mechanical, chemical, and biological indicators. In the present investigation, none of these monitoring procedures were implemented. Failure to perform routine sterilization validation substantially increases the risk of undetected sterilization failure and subsequent transmission of pathogens through surgical instruments [25].

Similar outbreaks associated with contaminated sterilization systems have been reported in ophthalmic surgical facilities. Previous studies have demonstrated that water reservoirs and internal components of rapid-cycle sterilizers may harbor Gram-negative bacteria capable of forming biofilms [26]. Even when organisms are partially inactivated during sterilization cycles, residual bacterial endotoxins or incomplete sterilization may contribute to postoperative complications. These findings highlight the critical importance of appropriate maintenance, reservoir drainage, and routine monitoring of sterilization systems used in high-volume surgical settings.

The detection of *P. aeruginosa* in environmental drains further suggests that widespread environmental contamination may have been present within the facility. Hospital plumbing systems, including sinks and drains, are recognized reservoirs for opportunistic pathogens and may contribute to environmental dissemination [27]. Although the PFGE similarity between drain isolates and patient isolates was lower than that observed for the sterilizer isolate, these sites may still have played a role in maintaining environmental contamination within the clinic.

This outbreak also illustrates the challenges of infection prevention and control in small outpatient surgical facilities. Unlike large hospitals, private clinics may lack structured infection control programs, standardized protocols, and dedicated personnel responsible for sterilization management. High surgical throughput combined with limited resources may increase the risk of deviations from recommended practices. The findings of this investigation emphasize that even routine procedures such as cataract surgery require strict adherence to sterilization standards and continuous monitoring to ensure patient safety.

Although the sterilizer was identified as the most probable source, other potential transmission pathways should also be considered. Healthcare workers may serve as transient or persistent reservoirs of pathogenic microorganisms, contributing to transmission within healthcare settings. Colonization, particularly on the hands, can facilitate the spread of healthcare-associated pathogens [28], and colonization with multidrug-resistant organisms is associated with an increased risk of subsequent infection and transmission [29]. Although no healthcare personnel tested positive for *P. aeruginosa* in this investigation, the potential implications of colonization among healthcare workers warrant consideration. Management would require a balanced approach incorporating infection control principles, occupational health considerations, and ethical safeguards.

In general, colonization alone does not necessarily indicate a direct source of transmission; however, in outbreak settings, temporary exclusion from high-risk procedures, reinforcement of hand hygiene and aseptic practices, and repeat microbiological testing may be considered. Any such measures should be implemented in accordance with institutional policies and public health guidance, where available. Importantly, management of colonized healthcare personnel must ensure strict confidentiality and avoid stigmatization, as unnecessary disclosure may have professional and psychological consequences. In the absence of standardized guidelines for such scenarios in outpatient surgical settings, decisions should be made on a case-by-case basis, guided by risk assessment, microbiological findings, and the potential for patient harm. These considerations highlight the need for clearer guidelines addressing the management of healthcare personnel colonization in outbreak situations, particularly in small ambulatory care settings.

Several limitations should be considered when interpreting the findings of this investigation. First, the investigation period was limited to October 2025, which restricted the ability to evaluate longer-term patterns of environmental contamination. Second, although several infection control deficiencies were identified, detailed analysis explaining why infections occurred exclusively among patients operated on 23 October was limited. The clustering of cases on a single surgical day suggests that a transient contamination event or a critical microbial load may have occurred within the sterilizer system. Third, additional sampling of internal sterilizer components was not performed, which might have provided further insight into the precise contamination pathway. Lastly, this investigation has inherent limitations related to its study design. In the absence of a control group, causal inference is limited, and the findings should be interpreted with caution. Although the convergence of epidemiologic, microbiologic, and environmental evidence strongly suggests a common source, the lack of a formal comparison group precludes definitive attribution of causality. In addition, inclusion of clinically diagnosed cases without microbiological confirmation may introduce misclassification; however, all cases in this investigation were microbiologically confirmed.

Despite these limitations, the convergence of epidemiologic, microbiologic, and environmental findings provides strong evidence that the sterilizer and instrument reprocessing system were the most likely source of this outbreak. These findings underscore the importance of strict adherence to instrument cleaning and sterilization protocols, routine sterilization monitoring, and ongoing infection control education for healthcare personnel.

## 5. Conclusions

This outbreak of postoperative endophthalmitis at Clinic A was most likely caused by contamination of the short-cycle steam sterilizer, not due to intrinsic device malfunction but rather due to inadequate infection prevention and control practices. Deficiencies in instrument cleaning, sterilization monitoring, environmental management, and staff training were identified as critical contributing factors. To prevent recurrence, it is essential to foster a patient safety-centered culture, mandate infection control education for surgical personnel, strengthen regulatory oversight of sterilization practices, and consider establishing a surveillance system for endophthalmitis cases through referral hospitals. Proactive detection and rapid response mechanisms are crucial to preventing healthcare-associated outbreaks in ophthalmic surgical settings. Outbreak investigations in ambulatory surgical settings remain underreported, particularly in small private clinics. This study highlights the importance of systematic infection control oversight in high-volume ophthalmic surgery environments.

## Figures and Tables

**Table 1:** Environmental Sampling Sites and Specimens Collected at Clinic A, October 2025.

No.	No. of Samples	Location	Collected Specimens
1	6	First-floor Examination Room (Director’s Office)	Microscope; tonometer; sink drain; astigmatism measurement device; desk surface; ophthalmic eye drops
2	1	First-floor Examination Room (Vice Director’s Office)	Microscope; desk surface
3	1	First-floor Injection Room Shelf	Opened lidocaine 2% vial
4	5	First-floor Diagnostic Room	Retinal imaging device; optic nerve imaging device; sink drain; infrared therapy device; autorefractor
5	4	Second-floor Laundry Room and Storage Area	Mop storage water; drain; blue mop; distilled water inlet of sterilizer No. 1
6	7	Second-floor Local Anesthesia Preparation Room	Pre-sterilization supplies; distilled water inlet; drain; sterile distilled water; floor; gauze container; towel
7	13	Second-floor Operating Room	Operating microscope; opened ophthalmic solutions (3 types); eyelash scissors; control buttons; surgical chair; nonwoven wipes; dehumidifier; green towel; floor; sterile distilled water; opened lidocaine 2% vial
8	5	Second-floor Operating Room Steam Sterilizer	Distilled water inlet; interior of cassettes No. 2 and No. 3; non-sterile instrument set No. 2
9	3	Second-floor Nurse Station Refrigerator	Opened hyaluronic acid syringe; alcohol swabs (plastic packaging); interior surface of refrigerator

## Data Availability

The data used in this work are available from the corresponding author upon reasonable request.

## Abbreviations

The following abbreviations are used in this manuscript:

PFGE	pulsed-field gel electrophoresis
KDCA	Korea Disease Control and Prevention Agency
TSA	Tryptic Soy Agar
